# Evaluation of bias in HIV seroprevalence estimates from national household surveys

**DOI:** 10.1136/sti.2008.030411

**Published:** 2008-07-22

**Authors:** V Mishra, B Barrere, R Hong, S Khan

**Affiliations:** Macro International Inc, Calverton, Maryland, USA

## Abstract

**Objectives:**

To evaluate HIV seroprevalence estimates from demographic and health surveys (DHS) and AIDS indicator surveys (AIS) for potential bias because of non-response and exclusion of non-household population groups.

**Methods:**

Data are from 14 DHS/AIS surveys with HIV testing, conducted during 2003–6. Blood samples were collected and analysed for HIV using standard laboratory and quality control procedures. HIV prevalence among non-tested adults was predicted based on multivariate statistical models of HIV for those who were interviewed and tested, using a common set of predictor variables. Estimates of the size of non-household populations in national censuses were used to assess potential bias because of their exclusion in the household surveys under different assumptions about proportion of adults and HIV prevalence in non-household populations.

**Results:**

Non-tested men had significantly higher predicted HIV prevalence than those tested in eight of the 14 countries, while non-tested women had significantly higher predicted prevalence than those tested in seven of the 14 countries. Effects of non-response were somewhat stronger in lower-prevalence countries. The overall effect of non-response on observed national HIV estimates was small and insignificant in all countries. Estimated effects of exclusion of non-household population groups were generally small, even in concentrated epidemics in India and Cambodia under the scenario that 75% of the non-household population was adults having 20 times greater HIV prevalence than adults in household surveys.

**Conclusions:**

Non-response and the exclusion of non-household population groups tend to have small, insignificant effects on national HIV seroprevalence estimates obtained from household surveys.

In countries with generalised epidemics, national estimates of HIV prevalence levels and trends in the adult population are generally derived indirectly from HIV surveillance among pregnant women attending selected antenatal clinics.^1^ ^2^ Recently, HIV seroprevalence data have also been collected in national population-based surveys, such as the demographic and health surveys and AIDS indicators surveys.^3^ These surveys have enabled direct estimates of population HIV prevalence.^4^ ^5^

A major challenge for the surveys is potential bias as a result of non-response.^4^ ^6^^–^8 Some eligible respondents may be absent at the time of the survey while others may be incapacitated or refuse to participate. The survey estimates of HIV prevalence may be biased to the extent that non-responders have different HIV prevalence levels than the responders. There is much evidence that mobility, which is one of the reasons for absence at the time of the survey, tends to be associated higher-risk sexual behaviours^9^^–^11 and risk of sexually transmitted infections,^12^ ^13^ including HIV infection.^9^^–^11 14 But some studies have failed to find an association between mobility and risk of HIV infection.^15^ ^16^ There is limited, inconclusive research on how refusal to participate in population-based surveys is associated with risky sexual behaviours.^6^ ^17^ In a recent study that included an assessment of non-response bias in five countries, Mishra *et al*^4^ concluded that non-responders tend to have somewhat higher HIV prevalence, but this bias has no significant effects on national seroprevalence estimates. Other previous studies have also failed to establish that population-based surveys significantly downwardly bias national HIV seroprevalence estimates.^17^^–^19

Another major challenge for the surveys is potential bias because of the exclusion of non-household population groups. Survey estimates may be biased to the extent that people residing in institutions (such as brothels, prisons, hostels, military/police barracks, long-term care homes) or those who are homeless have different HIV prevalence levels than those living in households and included in the survey sample. While there is considerable evidence that some of the institutional populations (such as brothels^20^ ^21^ and prisons^22^ ^23^) and the homeless^24^ tend to have higher risk of HIV infection, there is no previous empirical research to examine how exclusion of non-household population groups might impact national prevalence estimates based on household samples.

In this study, we expand the analysis of non-response bias in HIV seroprevalence estimates to 14 demographic and health surveys (DHS) and AIDS indicator surveys (AIS). Additionally, in five surveys with varying levels of HIV prevalence, we evaluate potential bias in national seroprevalence estimates because of exclusion of non-household population groups.

## METHODS

This study uses data from 14 nationally representative surveys of adult women and men, conducted during 2003 and 2006. Eleven of these surveys were DHS: Burkina Faso, Cambodia, Cameroon, Ethiopia, Ghana, India, Kenya, Lesotho, Malawi, Rwanda, Zimbabwe; and three were AIS: Cote d’Ivoire, Tanzania, Uganda. All these surveys included HIV testing and HIV serostatus data were linked to respondents’ socioeconomic and behavioural characteristics. Dried blood spot samples were collected (venous blood in Uganda) and analysed for HIV using standard laboratory and quality control procedures and internationally accepted ethical standards.^25^ HIV test results were linked anonymously to the characteristics and behaviours of the survey respondents.

In most surveys, nationally representative samples of women age 15–49 and men age 15–59 were tested for HIV. The only exceptions are Uganda where women age 15–59 were tested; Tanzania, Cote d’Ivoire and Cambodia where men age 15–49 were tested, and India, Kenya, Malawi and Zimbabwe where men age 15–54 were tested. In the 14 countries included in this analysis, the numbers eligible for HIV testing ranged from 3305 males (15–59) and 3758 females (15–49) in Lesotho to 64 175 males (15–54) and 62 182 females (15–49) in India.

### Analysis of bias because of non-response

To estimate the extent of non-response bias and its potential impact on the observed HIV rates in the 14 countries with linked data, all eligible respondents were divided into four groups: (1) interviewed and tested; (2) not interviewed but tested; (3) interviewed, not tested; and (4) not interviewed, not tested. Eligibility for individual interview and HIV testing was based on de facto population.

To evaluate the effect of non-response bias on the survey estimates, HIV prevalence is predicted among non-responding adults (groups 3 and 4) based on multivariate models of HIV for those who were interviewed and tested (group 1), using a common set of predictor variables. A logistic regression model is used, after accounting for clustering in the survey design, to calculate predicted HIV prevalence separately for the “not interviewed, not tested” and “interviewed, not tested” groups. Predictions for the “not interviewed, not tested” group are based on a limited set of variables (only from the household questionnaire), but predictions for the “interviewed, not tested” group additionally use several individual sociodemographic and behavioural characteristics of the respondents, as collected in the survey (see footnotes to table 2).

**Table 2:** Predicted HIV prevalence among the non-respondents and adjusted HIV prevalence estimate for all eligible males and females, DHS/AIS countries with linked HIV testing data, 2003–6

Country	Observed HIV prevalence among tested respondents	Predicted HIV prevalence among non-tested respondents						Adjusted prevalence among all eligible respondents
		Interview status		Reason not tested			Total non-tested	
		Interviewed	Not interviewed	Refused	Absent	Other/ missing		
**Burkina Faso 2003**								
Male (15–59)	1.94	2.68	2.48	2.91	2.52	2.11	2.57*	2.02
Female (15–49)	1.83	3.56	2.30	3.71	2.35	2.78	3.15*	1.94
**Cambodia 2005**								
Male (15–49)	0.62	1.07	0.79	1.09	0.82	0.38	0.88	0.64
Female (15–49)	0.61	1.49	0.61	1.41	0.54	0.53	1.02	0.63
**Cameroon 2004**								
Male (15–59)	3.91	5.17	5.10	5.44	5.00	3.71	5.13*	4.04
Female (15–49)	6.75	8.73	8.24	8.72	8.87	7.12	8.51*	6.90
**Cote d’Ivoire 2005**								
Male (15–49)	2.86	3.39	3.21	3.22	3.48	2.29	3.29	2.98
Female (15–49)	6.40	6.89	7.73	7.15	7.93	6.05	7.29*	6.64
**Ethiopia 2005**								
Male (15–59)	0.92	1.44	1.23	1.44	1.30	0.87	1.34*	0.99
Female (15–49)	1.86	3.46	3.23	3.50	4.07	1.62	3.39*	2.06
**Ghana 2003**								
Male (15–59)	1.66	2.14	1.62	2.27	1.50	2.40	1.98	1.72
Female (15–49)	2.70	2.97	2.46	3.10	2.40	2.56	2.77	2.70
**India 2005–6**								
Male (15–54)	0.35	0.44	0.53	0.44	0.51	0.58	0.50*	0.38
Female (15–49)	0.22	0.25	0.32	0.24	0.30	0.36	0.28*	0.23
**Kenya 2003**								
Male (15–54)	4.71	4.47	5.81	4.83	5.54	4.28	5.11	4.81
Female (15–49)	8.70	6.82	9.24	7.19	8.00	7.59	7.46*	8.45
**Lesotho 2004**								
Male (15–59)	18.94	19.12	19.18	18.94	18.32	20.66	19.15	19.01
Female (15–49)	26.37	25.17	24.54	25.70	23.80	23.72	25.00	26.09
**Malawi 2004**								
Male (15–54)	10.23	9.53	11.37	9.44	12.74	9.31	10.20	10.22
Female (15–49)	13.32	12.14	12.68	12.02	13.01	13.28	12.24	12.99
**Rwanda 2005**								
Male (15–59)	2.24	3.00	3.16	4.42	2.87	3.26	3.09*	2.28
Female (15–49)	3.61	5.74	3.84	5.21	4.53	4.75	4.64	3.64
**Uganda 2004–5**								
Male (15–59)	5.15	3.88	4.52	3.87	4.41	5.16	4.28*	5.03
Female (15–59)	7.29	6.24	7.01	6.58	6.86	5.92	6.58*	7.22
**Tanzania 2003**								
Male (15–49)	6.26	6.84	7.38	6.99	7.37	5.45	7.04*	6.44
Female (15–49)	7.70	8.40	7.20	8.36	7.29	6.94	8.11	7.77
**Zimbabwe 2005–6**								
Male (15–54)	14.75	15.28	17.38	15.79	16.67	19.05	16.35*	15.28
Female (15–49)	21.12	19.90	21.38	20.06	21.48	20.71	20.51	20.97

*Significantly different from the observed prevalence at p<0.05.

Variables for predicting HIV prevalence in the “not interviewed, not tested” group included age, education, wealth index, residence and geographic region.

Additional variables for predicting HIV in the “interviewed, not tested” group included: marital status; childbirth in last five years (women only); work status; media exposure; ethnicity; religion; circumcision (men only); STI or STI symptoms in the last 12 months; alcohol use at last sex in the last 12 months; number of sex partners in the last 12 months; cigarette smoking/tobacco use; age at first sex; number of lifetime sexual partners; number of sexual partners in the last 12 months; condom use at last sex in the last 12 months; higher-risk sex (sex with a non-marital, non-cohabiting partner) in the last 12 months; knowledge of prevention methods (abstinence, being faithful and condom use); attitudes towards people living with HIV (PLHIV). Woman’s ability to negotiate safer sex with spouse; woman’s participation in household decision-making (women only); number of medical injections in the last 12 months; duration of stay in current place of residence; number of times slept away in the last 12 months (men only); away (from usual place of residence) for more than one month in the last 12 months (men only); and previously tested for HIV. The list of additional variables used varied slightly from country to country, depending on the availability of information.

Multivariate analyses used Stata version 9.0. Analysis was carried out separately for males and females for each country. Adjusted HIV prevalence was calculated as a weighted average of observed prevalence among those who were tested and predicted prevalence in the two groups of non-tested respondents. Sampling weights were applied in accordance with standard DHS procedures. We used HIV sampling weights for the tested, individual sampling weights for the “interviewed, not tested”, and household sampling weights for the “not interviewed, not tested” groups, respectively.

### Analysis of bias because of exclusion of non-household population

In five of the countries (Cambodia, India, Ghana, Uganda and Lesotho), we examine potential bias because of exclusion of non-household population groups on the survey estimates of HIV prevalence for adults age 15–49. These countries were chosen to represent countries at varying levels of HIV prevalence.

For this purpose, we obtained national estimates of the size of household population, size of non-household population (including both institutional and homeless), total population, the annual population growth rate and the proportion of adults age 15–49 in the total population in each country.^26^^–^30 Using the annual growth rate, the household, non-household and total population sizes were projected to the DHS survey year. Next, using the proportion of adults in the total population, numbers of adults in the household, non-household and total population were estimated for the survey year. Adults are more likely to live in institutions and be homeless than children or elderly, but information on the age structure of the non-household population was not readily available from census in most cases. We therefore used different assumptions about the proportion of adults in the non-household population and the level of HIV prevalence among non-household adults to estimate overall HIV prevalence among all adults in each country (accounting for exclusion of non-household population groups).

We estimated the potential impact of excluding non-household population groups under the following three scenarios:

- *Scenario A (baseline):* The proportion of adults (15–49) in the non-household population is the same as in the census population; and HIV prevalence among non-household adults is the same as the prevalence among adults in the household survey.
- *Scenario B:* The proportion of adults (15–49) in the non-household population is 66.67%; and the HIV prevalence among the non-household adults is 10 times in India and Cambodia, five times in Ghana, two times in Uganda, 1.5 times in Lesotho that of the prevalence among adults in the household survey.
- *Scenario C:* The proportion of adults (15–49) in the non-household population is 75.00%; and the HIV prevalence among the non-household adults is 20 times in India and Cambodia, 10 times in Ghana, four times in Uganda, two times in Lesotho that of the prevalence among adults in the household survey.

## RESULTS

HIV prevalence among adults (15–49) in the 14 countries ranged from less than 1% in India and Cambodia to 23.2% in Lesotho. Despite large HIV prevalence differences among the surveys, fairly consistent patterns of HIV infection are observed by age, sex and urban/rural residence (data not shown).

### Estimates of bias because of non-response

Household response rates were very high in all surveys (93% or higher) (table 1). Response rates for the individual interview were also above 90% in most surveys. Individual interview response rates for females ranged from 90% in Cote d’Ivoire and Zimbabwe to 98% in Rwanda. Individual interview response rates for males were lower than for females in all 14 countries, and ranged from a low of 82% in Zimbabwe to 97% in Rwanda.

**Table 1:** Response rates for household interview, individual interview and HIV testing by sex, and reason for HIV non-response, DHS/AIS countries with HIV testing, 2003–6

Country, sex (age)	Household response rate	Number eligible for individual interview and HIV testing	Individual response rate	HIV response rate	Reason for HIV non-response		
					Refused	Absent	Other/ missing
**Burkina Faso 2003**	99.4						
Male (15–59)		3984	90.5	85.8	5.9	5.1	3.2
Female (15–49)		4575	96.7	92.3	4.0	2.0	1.7
**Cambodia 2005**	98.0						
Male (15–49)		7229	93.1	90.3	3.7	5.1	0.9
Female (15–49)		8638	97.2	95.1	2.7	1.5	0.6
**Cameroon 2004**	97.6						
Male (15–59)		5676	93.0	89.8	5.2	3.5	1.5
Female (15–49)		5703	94.5	92.1	5.1	1.5	1.3
**Cote d’Ivoire 2005**	95.5						
Male (15–49)		5148	87.5	75.8	13.9	9.2	1.1
Female (15–49)		5772	89.8	78.7	14.5	5.2	1.6
**Ethiopia 2005**	98.6						
Male (15–59)		6778	89.0	75.4	15.0	7.8	1.9
Female (15–49)		7142	95.4	83.2	13.3	2.3	1.3
**Ghana 2003**	98.7						
Male (15–59)		5345	93.8	80.0	10.3	7.5	2.2
Female (15–49)		5949	95.7	89.3	5.3	3.7	1.8
**India 2005–6**	92.9						
Male (15–54)		64 175	86.5	78.1	6.9	12.1	3.0
Female (15–49)		62 182	93.6	85.0	8.1	4.5	2.4
**Kenya 2003**	96.3						
Male (15–54)		4183	85.5	70.3	12.8	13.9	2.9
Female (15–49)		4303	94.0	76.3	14.5	6.3	2.8
**Lesotho 2004**	97.4						
Male (15–59)		3305	84.6	68.0	16.6	8.7	6.8
Female (15–49)		3758	94.2	80.7	12.1	2.9	4.3
**Malawi 2004**	97.8						
Male (15–54)		3797	85.9	63.3	24.6	9.3	2.8
Female (15–49)		4071	94.9	70.4	24.0	2.5	3.1
**Rwanda 2005**	99.6						
Male (15–59)		4959	97.2	95.6	0.4	3.3	0.8
Female (15–49)		5837	98.1	97.3	0.3	2.0	0.5
**Tanzania 2003**	98.5						
Male (15–49)		6196	91.3	77.1	14.9	7.0	1.1
Female (15–49)		7154	95.9	83.5	12.9	2.8	0.8
**Uganda 2004–5**	96.8						
Male (15–59)		9905	89.1	83.8	5.8	8.8	1.6
Female (15–59)		11 454	94.5	89.3	5.2	4.0	1.6
**Zimbabwe 2005–6**	95.0						
Male (15–54)		8761	81.9	63.4	21.0	12.9	2.7
Female (15–49)		9870	90.2	75.9	15.3	6.4	2.3

Response rates for HIV testing were lower than those for individual interview in all cases. In seven of the 14 countries, the difference in the response rates for individual interview and for HIV testing was greater than 10 percentage points for both males and females. The highest differences were observed in Malawi, where the response rate for HIV testing was 23 percentage points lower for males and 25 percentage points lower for females than the corresponding response rates for individual interview. On the other hand, Rwanda had the smallest differences between the individual interview and HIV testing response rates of about 2 percentage points for males and 1 percentage point for females.

HIV response rates for males were lowest in Malawi and Zimbabwe (63%), followed by Lesotho (68%) and Kenya (70%). The highest male HIV response rates were in Rwanda (96%), followed by Cambodia and Cameroon (90% each). Similar to individual interview response rates, HIV response rates for females were considerably higher than for males in all countries. Female HIV response rates ranged from 70% in Malawi to 97% in Rwanda, and were above 90% also in Cameroon, Burkina Faso and Cambodia.

Refusal was a more important reason for HIV non-response than absence in all countries for women (except in Rwanda) and in nine of the 14 countries for men. In Rwanda, very few women or men refused testing. In all countries, men were much more likely than women to be absent for testing. In 12 of the 14 countries, the HIV non-response rate because of absence was two to four times greater for men than for women.

Non-response rates because of both refusal and absence were much higher in urban areas than in rural areas. Also, the non-response rates were considerably higher among more educated and wealthier respondents. In five of the eight countries, where data on chronically ill adults (seriously ill for three or more months in the past year) were available, response rates were slightly higher among chronically ill adults than among adults who were not chronically ill. There were no clear patterns in the HIV non-response rates by various risk and protective factors (data not shown).

In most countries, non-tested males and females have higher predicted HIV prevalence than the observed prevalence among those who were tested (table 2). In eight of the 14 countries for males and in seven of the 14 countries for females, the predicted prevalence among non-tested individuals is significantly greater than the observed prevalence among those tested. In Uganda for both males and females and in Kenya for females, the predicted prevalence among the non-tested individuals is significantly lower than among those tested.

**Figure 1:**
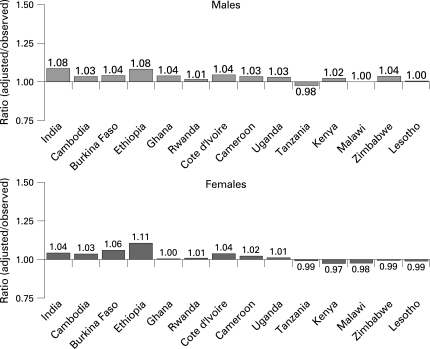
Ratios of adjusted HIV prevalence among all eligible individuals to observed HIV prevalence among those tested in the surveys

Adjusting the observed national HIV estimates from tested males and females by accounting for the predicted rates among the non-tested makes little difference to the observed estimates in most cases (fig 1). Even in countries where predicted prevalence among the non-responders is significantly higher or lower, the adjusted prevalence for all eligible respondents is about the same as the observed prevalence based only on the tested respondents. Although not statistically significant in all 14 countries, the effects of non-response tend to be somewhat greater among lower prevalence countries for both males and females.

### Estimates of bias because of exclusion of non-household population

Our simulation analyses for India, Cambodia, Ghana, Uganda and Lesotho show that under varying assumptions of much greater HIV prevalence among non-household adults, estimated bias because of exclusion of non-household population groups in national HIV prevalence estimates from household samples tends to be small (table 3).

**Table 3:** Potential effects of exclusion of non-household population on the national HIV estimate for adults age 15–49, India, Cambodia, Ghana, Uganda, Lesotho

	Projected population in survey year	% population (15–49)	Projected population (15–49)	HIV prevalence		No of HIV+ in population (15–49)	Estimated HIV prevalence
**India 2005–6**							
**Scenario A (baseline)**							
Population living in households	1 101 648 431	53.18%	585 804 092	Svy Est	0.28%	1 640 251	
Non-household population	10 538 569	53.18%	5 603 908	NHH% = Svy%	0.28%	15 691	
Total population	1 112 187 000	53.18%	591 408 000			1 655 942	0.28%
**Scenario B**							
Population living in households	1 101 648 431	53.05%	584 381 936	Svy Est	0.28%	1 636 269	
Non-household population	10 538 569	66.67%	7 026 064	NHH% = 10*Svy%	2.80%	196 730	
Total population	1 112 187 000	53.18%	591 408 000			1 832 999	0.31%
Scenario C							
Population living in households	1 101 648 431	52.97%	583 504 073	Svy Est	0.28%	1 633 811	
Non-household population	10 538 569	75.00%	7 903 927	NHH% = 20*Svy%	5.60%	442 620	
Total population	1 112 187 000	53.18%	591 408 000			2 076 431	0.35%
**Cambodia 2005**							
**Scenario A (baseline)**							
Population living in households	13 056 114	50.40%	6 580 282	Svy Est	0.62%	40 798	
Non-household population	271 833	50.40%	137 004	NHH% = Svy%	0.62%	849	
Total population	13 327 947	50.40%	6 717 285			41 647	0.62%
**Scenario B**							
Population living in households	13 056 114	50.06%	6 536 054	Svy Est	0.62%	40 524	
Non-household population	271 833	66.67%	181 231	NHH% = 10*Svy%	6.20%	11 236	
Total population	13 327 947	50.40%	6 717 285			51 760	0.77%
**Scenario C**							
Population living in households	13 056 114	49.89%	6 513 411	Svy Est	0.62%	40 383	
Non-household population	271 833	75.00%	203 875	NHH% = 20*Svy%	12.40%	25 280	
Total population	13 327 947	50.40%	6 717 285			65 664	0.98%
**Ghana 2003**							
**Scenario A (baseline)**							
Population living in households	18 382 204	48.60%	8 933 751	Svy Est	2.16%	192 969	
Non-household population	19 982	48.60%	9711	NHH% = Svy%	2.16%	210	
Total population	18 402 186	48.60%	8 943 462			193 179	2.16%
**Scenario B**							
Population living in households	18 382 204	48.58%	8 930 140	Svy Est	2.16%	192 891	
Non-household population	19 982	66.67%	13 322	NHH% = 5*Svy%	10.80%	1439	
Total population	18 402 186	48.60%	8 943 462			194 330	2.17%
**Scenario C**							
Population living in households	18 382 204	48.57%	8 928 476	Svy Est	2.16%	192 855	
Non-household population	19 982	75.00%	14 987	NHH% = 10*Svy%	21.60%	3237	
Total population	18 402 186	48.60%	8 943 462			196 092	2.19%
**Uganda 2004–5**							
**Scenario A (baseline**)							
Population living in households	26 172 937	42.30%	11 071 153	Svy Est	6.38%	706 340	
Non-household population	423 793	42.30%	179 264	NHH% = Svy%	6.38%	11 437	
Total population	26 596 730	42.30%	11 250 417			717 777	6.38%
**Scenario B**							
Population living in households	26 172 937	41.91%	10 967 874	Svy Est	6.38%	699 750	
Non-household population	423 793	66.67%	282 542	NHH% = 2*Svy%	12.76%	36 052	
Total population	26 596 730	42.30%	11 250 417			735 803	6.54%
**Scenario C**							
Population living in households	26 172 937	41.77%	10 932 572	Svy Est	6.38%	697 498	
Non-household population	423 793	75.00%	317 844	NHH% = 4*Svy%	25.52%	81 114	
Total population	26 596 730	42.30%	11 250 417			778 612	6.92%
**Lesotho 2004**							
**Scenario A (baseline)**							
Population living in households	1 866 354	50.90%	949 974	Svy Est	23.20%	220 394	
Non-household population	7913	50.90%	4028	NHH% = Svy%	23.20%	934	
Total population	1 874 267	50.90%	954 002			221 328	23.20%
**Scenario B**							
Population living in households	1 866 354	50.83%	948 726	Svy Est	23.20%	220 104	
Non-household population	7913	66.67%	5276	NHH% = 1.5*Svy%	34.80%	1836	
Total population	1 874 267	50.90%	954 002			221 940	23.26%
**Scenario C**							
Population living in households	1 866 354	50.80%	948 067	Svy Est	23.20%	219 952	
Non-household population	7913	75.00%	5935	NHH% = 2*Svy%	46.40%	2754	
Total population	1 874 267	50.90%	954 002			222 705	23.34%

NHH: non-household; Svy: survey; Est: estimate.

Census data on the size of total population, size of non-household population and proportion of adults in the national population were obtained from India 2001 Census, Cambodia 2004 Inter-Censal Survey, Ghana 2000 Census, Uganda 2002 Census, Lesotho 2006 Census.

In India, the actual proportion of non-household adults in the census was 56.15%, much lower than 66.67% and 75.00% assumed in scenarios B and C.

In Ghana, the estimate of non-household population refers to homeless population only.

In Lesotho, the estimate of non-household population refers to institutional population only.

In India, for example, under scenario B where the proportion of adults age 15–49 in the non-household population is assumed to be 67% and the HIV prevalence among non-household adults is assumed to be 10 times the prevalence among household adults (2.80%), the estimated HIV prevalence among all adults increases only slightly, from 0.28% to 0.31%. Under scenario C, where the proportion of adults in the non-household population is assumed to be 75% and the HIV prevalence among non-household adults is assumed to be 20 times the prevalence among household adults (5.60%), the estimated HIV prevalence among all adults increases to 0.35%. Similarly, in Cambodia, the observed HIV prevalence in the survey (0.62%) increases to 0.77% under scenario B and 0.98% under scenario C. In Ghana, Uganda and Lesotho, with much higher levels of HIV prevalence, estimated bias because of exclusion of non-household population groups tends to be relatively smaller.

## DISCUSSION

HIV response rates for females were considerably higher than for males in all countries. The lower response rates for males mainly reflect more frequent absence of men from the households. In 12 of the 14 countries, the HIV non-response rate because of absence was two to four times greater for males than for females. Non-response rates were higher among urban, more-educated and wealthier respondents. These patterns of non-response are typical of most household surveys in developing countries. However, there were no clear patterns in non-response rates by various risk and protective factors. Chronically ill adults were equally or more likely to participate in the surveys, suggesting that differential participation of chronically ill adults is unlikely to be a major source of bias.

The non-responder males and females tend to have higher predicted HIV prevalence than those tested. In eight of the 14 countries for males and in seven of the 14 countries for females, non-responders have significantly higher predicted prevalence, but consistent with previous research, the overall effects of non-response on the observed national HIV prevalence estimates are small and insignificant in all 14 countries.^4^ ^17^^–^19 The small effects of the non-response bias on the observed national estimates are due mainly to a much smaller proportion of non-responders than those who were tested in the surveys. The effects of non-response are somewhat greater among lower prevalence countries for both males and females.

Our analysis of potential bias in the national HIV prevalence estimates because of the exclusion of non-household population in five countries indicated that exclusion of non-household population groups in the surveys is likely to have only a minimal effect on the observed national HIV prevalence estimates. This bias is expected to be greater in countries with concentrated epidemics. Our analysis shows that even in countries with concentrated epidemics (for example, India with a survey HIV prevalence estimate of 0.28%), HIV prevalence in the non-household groups needs to be orders of magnitude higher for it to have any significant effect on the national estimate based on the household sample.

In the analysis of the non-response bias, a major limitation is that the estimates are only adjusted to the extent that the sociodemographic and behavioural characteristics included in the analysis are correlated with the risk of HIV infection. Despite including about 30 predictor variables in the regression models, only about 20% of variation in HIV prevalence is explained in most countries, indicating the limitation of such modelling in explaining behavioural health outcomes. Another limitation is that the adjustments for not interviewed, not tested respondents are based on limited information available from the household questionnaire. Future surveys should attempt to collect additional information on this group (mostly absentees) to better assess potential bias due to their exclusion.

Our analysis is based on de facto household-based sample of the national population. A de facto sample assumes that usual residents (de jure household members) who did not spend the previous night in their own household are, on average, interviewed in a household they may be visiting. A de facto sample maximises participation rates and avoids potential double counting of respondents. HIV seroprevalence estimates based on de facto samples may be biased to the extent some of the de jure household members who slept away may not be visiting another household and to the extent such people have differential HIV prevalence.

Furthermore, the adjustments for bias because of non-response and exclusion of non-household population groups do not account for a small proportion (usually 1–3%) of sampled households that were not interviewed in the surveys. Finally, the assumptions regarding HIV prevalence and the proportion of adults in the non-household population are arbitrary. However, in India where information on the age structure of non-household population was available from the census, the proportion of adults (15–49) in the non-household population was much lower (56%) than the assumed levels of 67% and 75% in the analysis. Moreover, because males tend to have lower prevalence than females and because a great majority of the institutional and homeless population tends to be males, our assumptions of 10 and 20 times greater prevalence among non-household adults seems reasonable.

Our analyses suggest that population-based surveys provide reliable, nationally representative direct estimates of HIV seroprevalence in countries with generalised epidemics. HIV prevalence data from population-based surveys can be useful in understanding the magnitude and spread of the epidemics and in calibrating estimates from sentinel surveillance.

**Key messages**

- Non-response in national household surveys tends to have small, not significant effects on HIV seroprevalence estimates based on tested respondents.
- Exclusion of institutional and homeless population in the surveys is likely to have only a minimal effect on national HIV seroprevalence estimates from household surveys.
- Population-based surveys provide reliable, nationally representative direct estimates of HIV seroprevalence in countries with generalised epidemics.
